# The Oncology Safety of Diagnostic Hysteroscopy in Early-Stage Endometrial Cancer: A Systematic Review and Meta-Analysis

**DOI:** 10.3389/fonc.2021.742761

**Published:** 2021-10-21

**Authors:** Yi Du, Yu Xu, Zhaojuan Qin, Liang Sun, Yali Chen, Ling Han, Ai Zheng

**Affiliations:** ^1^ Department of Obstetrics and Gynecology, West China Second University Hospital, Sichuan University, Chengdu, China; ^2^ Department of Pathology, West China Second University Hospital, Sichuan University, Chengdu, China; ^3^ Key Laboratory of Birth Defects and Related Diseases of Women and Children, Sichuan University, Ministry of Education, Chengdu, China

**Keywords:** hysteroscopy, endometrial cancer, early-stage, oncology, meta-analysis, systematic review

## Abstract

**Background:**

Hysteroscopy is becoming a common method for the diagnosis of uterine disorders in developed countries. However, hysteroscopy might worsen the prognosis of endometrial cancer because it could cause cancer dissemination into the peritoneal cavity through the fallopian tubes. Objective: The aim of this systematic review and meta-analysis was to explore the oncological safety of hysteroscopy for early-stage endometrial cancer.

**Search Strategy:**

Eligible studies were obtained from PubMed, Embase, and the Cochrane Library up to September 22, 2020.

**Selection Criteria:**

Studies which compared the oncological safety of hysteroscopy with other methods were included.

**Data Collection and Analysis:**

A total of 3980 patients were included in this study, of whom1357 patients had undergone hysteroscopy and2623 had not.

**Main Results:**

There was no significant association between hysteroscopy and worse prognosis in early-stage endometrial cancer [disease-free survival: log risk ratio(logRR) -0.22; 95% confidence interval (CI), -0.54 to 0.1; p=0.97; overall survival: logRR 0.03; 95% CI, -0.05 to 0.11; p=0.02; disease-specific survival: logRR 0.03; 95% CI, -0.03 to 0.10; p=0.00].

**Conclusion:**

This study suggests that hysteroscopy is a safe diagnostic and treatment method, and has no significant effect on the prognosis of early-stage endometrial cancer.

**Systematic Review Registration:**

PROSPERO registration number: CRD42020193696.

## Introduction

Endometrial cancer is a gynecological malignancy that is common in developed countries. The incidence and mortality rate of endometrial cancer have been rising in recent years (1, 2). In 2020, an estimated 65,620 new cases of endometrial cancer were diagnosed, and more than 12,000 deaths occurred in the United States owing to this malignancy, whereas 61,380 were diagnosed and 10,920 deaths were recorded in 2017 (2, 3). Fortunately, most patients diagnosed with early-stage endometrial cancer show a good prognosis after surgery alone.

Abnormal uterine bleeding (AUB) is the main symptom of endometrial cancer, in which postmenopausal bleeding accounts for approximately 90% (4). The American College of Obstetricians and Gynecologists (ACOG) recommends transvaginal ultrasound (TVU) as the appropriate initial test for postmenopausal bleeding when the thickness of endometrial echo is less than 4mm. When TVU fails to identify a thin endometrial echo in a postmenopausal woman with bleeding, a tissue sample should be evaluated (5). In the US and Europe, dilatation and curettage (D&C) has traditionally been the preferred endometrial sampling method. However, there are many disadvantages with D&C, such as its blind nature and the requirement for general anesthesia. Currently, hysteroscopy is considered the gold standard in uterine cavity evaluation because it can not only provide direct visualization of the uterine cavity but can be combined with focal biopsy or curettage (6). A systematic review reported that the diagnostic accuracy of hysteroscopy for endometrial cancer was high, with a sensitivity and specificity of 86.4% and 99.2% respectively (6, 7). However, hysteroscopy may cause endometrial cancer cells to migrate into the peritoneal cavity through the fallopian tubes in endometrial cancer. In a meta-analysis by Polyzos et al. (8), hysteroscopy increased the risk of cancer cell dissemination into the peritoneal cavity in patients with endometrial cancer. Gurkan et al. (9) suggested that malignant cells disseminating into the abdominal cavity from the uterus could be functionally viable and adhere to a matrix. These functionally viable malignant cells may become metastatic, leading to recurrence of disease or even death.

Although hysteroscopy appears to be able to disperse possibly functionally viable tumor cells into the peritoneal cavity, many studies found that hysteroscopy or positive peritoneal cytology (PPC) did not negatively affect the prognosis of endometrial cancer (10, 11). However, most of these studies were retrospective, with samll sample sizes. Thus, evidence from these studies is not robust enough to draw definitive conclusions. To the best of our knowledge, many systematic reviews andmeta-analyse have been performed on the association between PPC and hysteroscopy in endometrial cancer, but there are no studies on the impact of hysteroscopy for prognosis. Therefore, in this study we performed a systematic review and meta-analysis to evaluate the oncological safety of hysteroscopy in early-stage endometrial cancer.

## Methods

The systematic review and meta-analysis were performed according to the Cochrane Handbook for Systematic Reviews of Interventions (12) and was presented based on the Preferred Reporting Items for Systematic Reviews and Meta-analyses guidelines (13). The protocol for this meta-analysis is available in PROSPERO (CRD42020193696).

### Search Strategy

The PubMed, Embase, and Cochrane Library databases were searched by two independent investigators (YD and YX) up to September 22, 2020. The search strategy included the Medical Subject Headings (MeSH) and text words for the following terms: “endometrial cancer”, “hysteroscopy”, “randomized controlled trial”, and “cohort study” (**Supplementary Table 1**). References from all included studies were cross-checked for additional relevant studies (7).

### Selection Criteria

Two independent reviewers (YD and ZQ) perused the retrieved abstracts and titles of the studies that were eligible for inclusion. When a decision could be made, full-text screening was done to make the final decision. When an agreement could not be reached, a third independent reviewer (AZ) was consulted to resolvethe differences.

Studies were included if they met the following inclusion criteria: (1) original studies reporting survival effects of diagnostic hysteroscopy on endometrial cancer *versus* non-hysteroscopy; (2) studies enrolling patients with early-stage endometrial cancer, including stages I and II under the FIGO 2009 staging criteria, or stages I to IIIA (only including PPC) under FIGO 1988 staging criteria; and (3) studies published in peer-reviewed English journals. The exclusion criteria were as follows: (1) case reports, case series, letters, commentaries, notes, and editorials; and (2) non-human trials.

### Data Extraction

Data were extracted from eligible studies by two independent investigators (YD, YC), including the study title, first author’s name, publication years, country of origin, participant characteristics, duration of follow-up, number of participants in each group, histologic type, grade, stage, primary outcomes:disease-free survival (DFS) and secondary outcomes:overall survival (OS) and disease-specific survival (DSS). DFS was considered as the period from the date of surgery to the date of first recurrence. OS was calculated from the date of surgery to the date of death. DSS was defined as the duration from the date of surgery to the date of death related to endometrial cancer. When the endometrial cancer was staged using the FIGO 1988 staging system, IIIA stage (including only (PPC) was classified as early-stage cancer.

### Quality Assessment

The methodological quality of the eligible studies was assessed by two independent researchers (YD and LS) according to the Cochrane risk of bias (ROB) criteria for randomized controlled studies (RCTs) and the Newcastle-Ottawa Quality Assessment Scale (NOS) for non-randomized studies. RCT studies were graded as ‘low risk’, ‘high risk” or “some concerns”, according to the following five domains: randomization process, deviations from intended interventions, missing outcome data, measurement of the outcome, and selection of the reported result (12). Each non-randomized study was judged according to the NOS by a star system using the following three domains: selection, comparability, and outcome for cohort studies or exposure for case-control studies. Studies with 7 or more stars in total were of high quality, studies with 6 stars were of moderate quality, and studies with less than 6 stars were of low quality (14).

### Statistical Analysis

Data were pooled using a random-effects models and log risk ratios (LogRR), and 95% confidence intervals (CIs) were calculated using DerSimonian and Laird modeling. Statistical heterogeneity between summary data was evaluated using I^2^ and P statistics. If P < 0.1, or I^2^ ≥ 50%, studies were considered to have significant heterogeneity, and sensitivity analysis was performed by excluding low-quality studies. A balanced funnel plot was used to identify publication bias. In circumstances where there was too much heterogeneity, ‘narrative synthesis’ was conducted. STATA-16 (version 16.0; StataCrop, College Station, TX) was used to perform the meta-analysis.

The statistical heterogeneity in the meta-analysis for 5-year OS (p=0.02, I^2^ = 80.15%) and DSS (p=0.00, I^2^ = 93.08%) was significant. Thus, this study did not conduct further analyses to detect heterogeneity.

## Results

### Characteristics of Retrieved Studies

**Figure 1:**
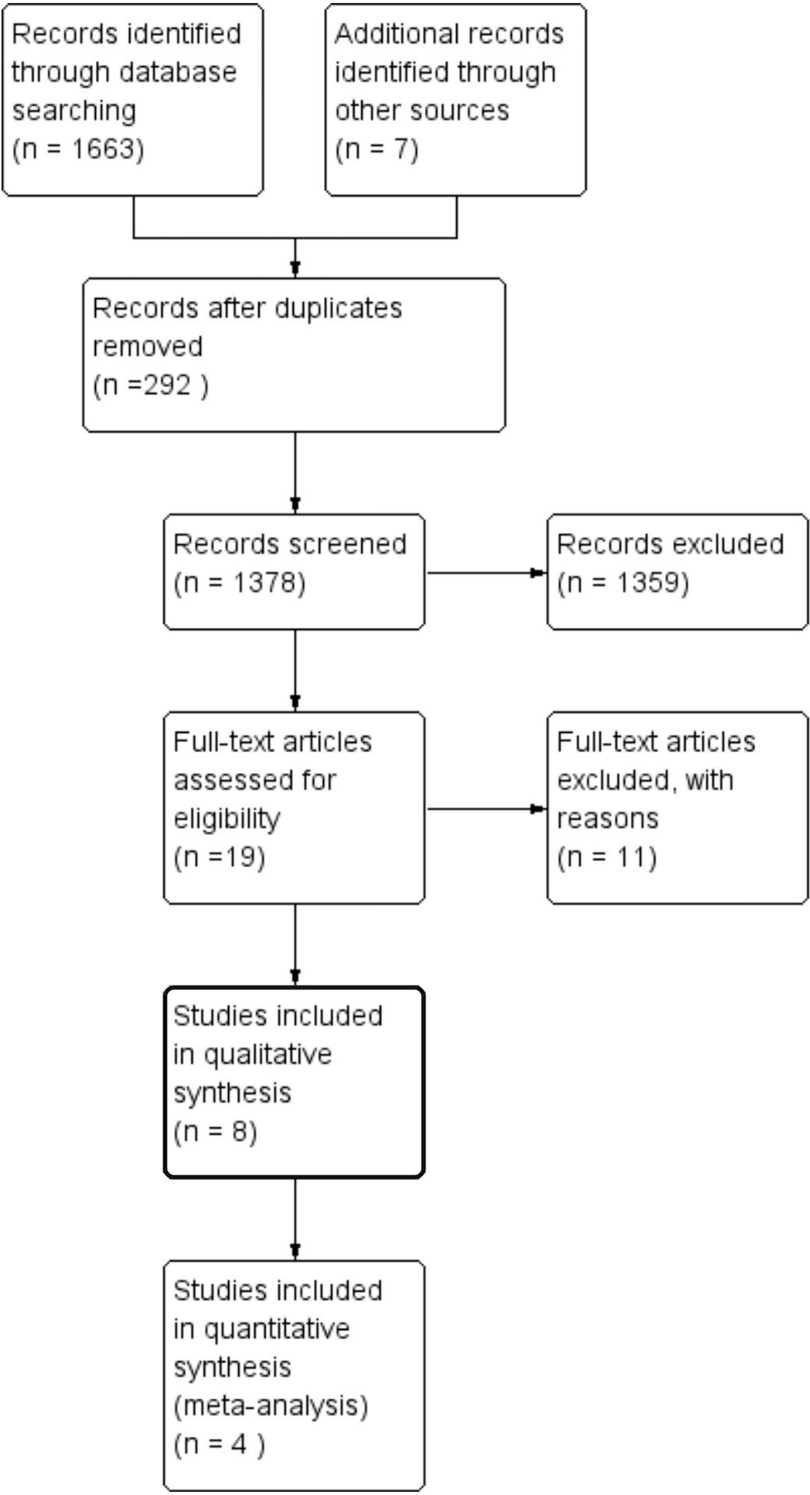
Study selection flow chart.

A total of 1670 potentially eligible records were retrieved from the literature search and additional relevant studies from references. The titles and abstracts of the records were screened according to inclusion and exclusion criteria. After full-text review, eight articles including two RCTs (15, 16) and six cohort studies (17–22) were eventually included in this study (**Figure 1**).

There were 3980 participants included in this study, of whom 1357 (34%) underwent hysteroscopy and 2623 (66%) did not. There were 190 (5%) patients in RCTs and 3790 (95%) in retrospective studies. Among the two RCTs, 100 (53%) patients underwent hysteroscopy, while 1257 (33%) patients underwent hysteroscopy in the six retrospective studies.

The mean age of patients which was mentioned in four studies ranged from 64.7 to 68.1 years. The duration of follow-up was reported in six of eight studies and ranged from 1.32 to 153 months. PPC was reported in seven studies, ranging from 2.6% to 27.5% in the hysteroscopy group, and from 0.7% to 18.2% in the non-hysteroscopy group. Four articles mentioned adjuvant therapy in early-stage endometrial cancer and none of them described significant difference in postoperative adjuvant treatment between groups. The detailed characteristics of the retrieved studies are listed in **Table 1**.

**Table 1:** Characteristics of Included Studies.

Auther (year)	type	H	NH	FIGO stage	mean age (SD)			Histologic Grading (%)			Histologic subtype (%)			Positive cytology (%)		adjuvant treatment (%)			Median follow-up (months)	Quality Evaluation
					H	NH	TATAL	GRADE	H	NH	subtype	H	NH	H	NH	type	H	NH		
1-Namazov et al. (2019) (17)	Retrospected cohort study (article)	355	969	I	64.7(10.3)	65.6(10.8)	65.4(10.7)	LG	261(74.4)	722 (74.7)	LG	261(74.4)	722 (74.7)	8	2.1	CT	116 (32.7)	327 (33.7)	52(12-120)	High (8)
								HG	71 (20.2)	227 (23.5)	HG	71 (20.2)	227 (23.5)							
																RT	32 (9.7)	101 (11.1)		
								Others	19 (5.4)	17 (1.8)	Others	19 (5.4)	17 (1.8)							
2-Chen et al. (2017) (18)	Retrospected cohort study (article)	40	59	I-II	/	/	/	/	/	/	type 1	0	0	27.5	5	/	/	/	/	High (8)
											Type2	100	100							
3-Aguilar et al. (2015) (19)	Retrospected cohort study (poster)	28	11	I-II	/	/	/	G1+G2	26(92.8)	7(63.7)	type 1	82.7	72.7	25	18.2	/	/	/	15	High (7)
								G3	2(7.2)	4(36.3)	type2	17.3	27.3							
4-Soucie et al. (2012) (20)	Retrospected cohort study (article)	621	1215	I-II	/	/	/	/	/	/	type 1	100	100	/	/	/	/	/	/	High (8)
											type2	0	0							
5-Cicinelli et al. (2010) (15)	Randomized contral study (article)	70	70	I-IIIA	66(16)	65(12)	65.5(14.1)	G1	43 (61.5)	27 (38.5)	type 1	100	100	5.7	8.6	BT	27 (19.2%)		62(2-123)	Low Risk
																BT+RT	32 (22.8%)			
								G2	27 (38.5)	25 (35.8)	type2	0	0							
																CT+RT	10 (7.1%)			
6-Monegat et al. (2009) (21)	Retrospected cohort study (poster)	78	152	I	/	/	/	/	/	/	type 1	100	100	2.6	3.3	/	/	/	69.23 (1.13–153)	High (7)
											type2	0	0							
7-de la Cuesta et al. (2004) (16)	Randomized contral study (article)	30	20	I-IIIA	68.1(10.2)	63(7.5)	66(9.5)	G1	6(30)	13(43)	type 1	100	100	5	10	WPR+BT	9	5	34(1.3-71.7)	Low Risk
								G2	12(60)	15(50)	type2	0	0			WPR	1	3		
								G3	2(10)	2(7)						BT	5	6		
8-Obermair A (2000) (22)	Retrospected cohort study (article)	135	127	I-IIIA	64.9(34-93)		64.9(7.5)	G1	62 (49.1)	64 (50.9)	type 1	52.2	47.8	3.7	0.7	None	52 (45.6)	62 (54.4)	23	Medium (6)
								G2	57 (56.4)	44 (43.6)						RT	42 (56.8)	32 (43.2)		
											type2	47.7	52.5							
								G3	11 (40.7)	16 (59.3)						CT	28 (71.8)	28.2)		

1. Studies after 2009 (17–20) used the FIGO 2009 staging criteria and trials before 2009 used 1988 staging criteria.

2. RCT studies were graded as ‘Low’, or ‘High’ risk of bias or’some concerns’; retrospected studieswith cumulative scores ≥7 stars were considered high quality, 6 stars medium quelity and less than 6 stars low quality.

3. H means hysterscopy group; NH means non-hysteroscopy group; PPC means positive peritoneal cytology; LG means low grade (endometroid grade 1–2, and villoglandular) ; HG means high grade (endometroid grade 3, uterine serous papillary carcinoma, clear cell carcinoma, and carcinosarcoma);G1,G2,G3 means endometrioid grade 1, grade2, grade3; PPC means positive peritoneal cytology; CT means Chemotherapy,RT means radiotherapy, BT means brachytherapy, WPR means whole pelvic radiotherapy, None means no adjuvant therapy.

4. type 1 endometrial cancer are of endometrioid histology, type 2 endometrial cancer include a variety of histologies such as clear cell, serous,carcinomas, squamous and/or undifferentiated carcinoma.

### Result of Quality Assessment

Eight studies were included in this study, including two RCTs (15, 16) and six retrospective studies (17–22). Both RCTs (15, 16) described an adequate random sequence generation process and were classified as low risk (15, 16). The median value of the NOS quality assessment for the six retrospective studies (17–22) was 7.5, with a mean score of 7.33 ± 0.745, and a range from 6 to 8. According to the NOS, five retrospective studies (17–21) were considered high quality, and one (22) was considered medium quality (**Table 1**).

### Primary Outcomes

**Figure 2:**
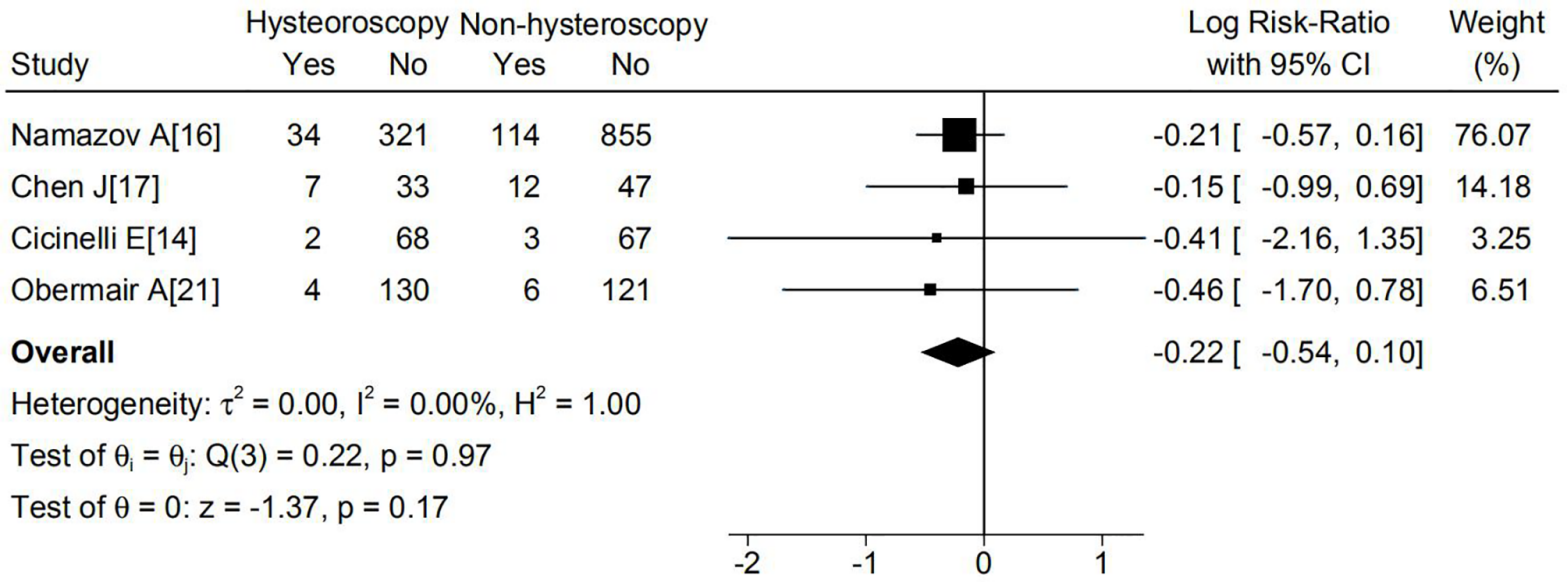
Effect of hysteroscopy on DFS in patients with or without hysteroscopy.

Seven studies reported DFS in total. Six of the these studies (15–19, 21, 22) reported that the hysteroscopy group had a higher DFS, and only one trial reported the opposite (21). None of the seven studies suggested a statistically worse DFS in patients who underwent hysteroscopy. A meta-analysis of the four studies based on 5-year DFS also suggested no significant difference between the hysteroscopy and non-hysteroscopy groups (logRR -0.22; 95% CI, -0.54 to 0.1; p=0.97) (**Figure 2**). Because P > 0.1, and I^2^ ≤ 50%, statistical heterogeneity and sensitivity analyses were not performed. The funnel plot was balanced, and no publication biased was observed (**Supplementary Figure 1**).

### Secondary Outcomes

**Figure 3:**
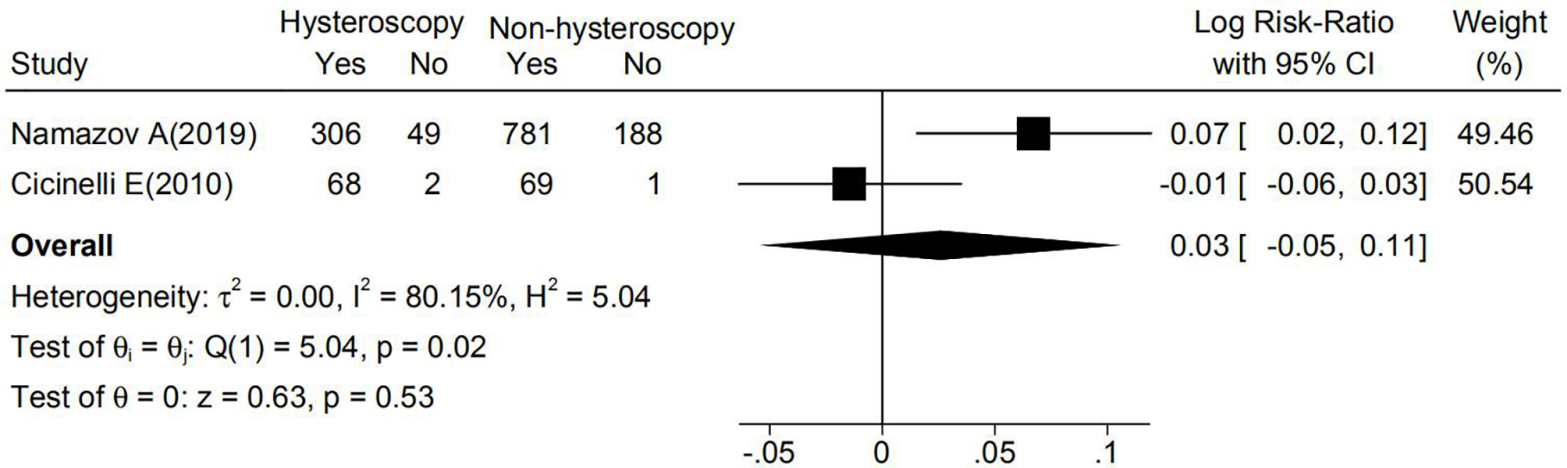
Effect of hysteroscopy on OS in patients with or without hysteroscopy.

There were no statistically significant differences in OS or DSS between the hysteroscopy and non-hysteroscopy groups in any study. Among five studies (15, 17, 19–21) reporting OS, two studies (17, 21) reported a higher OS in the hysteroscopy group, two studies (15, 20) reported a higher OS in the non-hysteroscopy group, and one study (19) reported no cases of endometrial cancer-related mortality. As shown in **Figure 3**, a meta-analysis of two studies based on 5-year OS suggested no significant difference between the two groups (logRR 0.03; 95% CI, -0.05 to 0.11; p=0.02).

**Figure 4:**
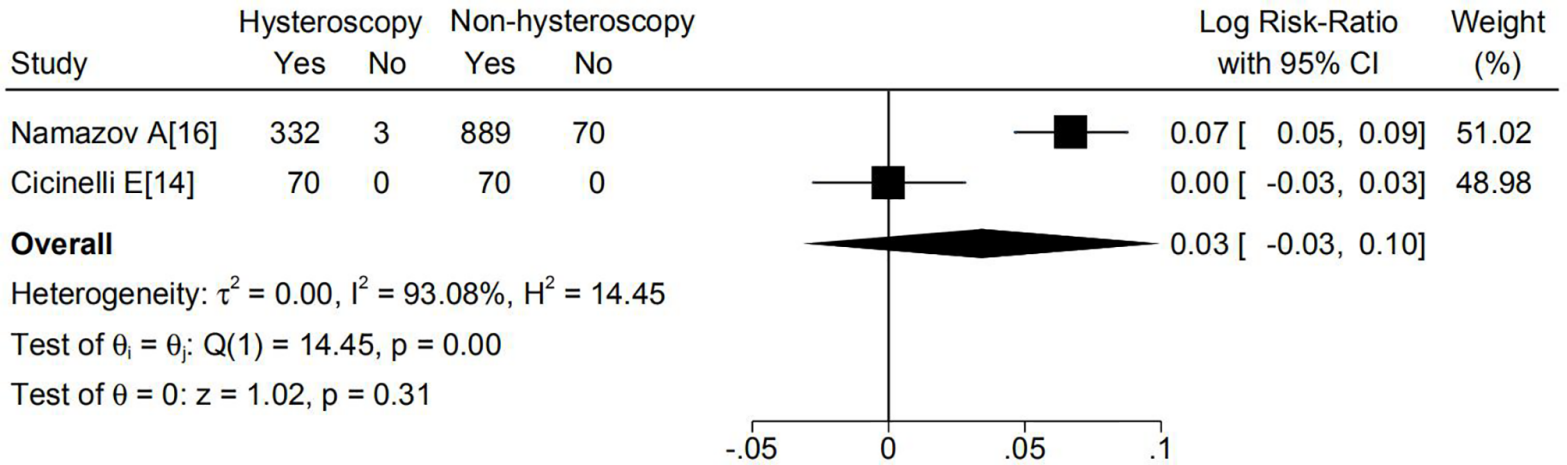
Effect of hysteroscopy on DSS in patients with or without hysteroscopy.

DSS was reported in four studies and only Chen et al. (19) observed higher DSS in the hysteroscopy group. As illustrated in **Figure 4**, there were no significant differences based on 5-year DSS between the two groups (logRR 0.03; 95% CI, -0.03 to 0.10; p=0.00).

## Discussion

Whether hysteroscopy is associated with a worse prognosis in early-stage endometrial cancer remains controversial. This study assessed the prognosis of patients with endometrial cancer undergoing hysteroscopy compared to patients who did noty. A meta-analysis was conducted for DFS, OS, and DSS. The results of this study suggest that hysteroscopy is not associated with a worse prognosis in early-stage endometrial cancer.

Malignant cells appearing in the peritoneal cavity in early-stage endometrial cancer might be the result of tumor dissemination through the fallopian tubes, extension *via* nearby lymphatics, or implantation metastasis from other extrauterine sites (23). Hysteroscopy is one of the causes of cancer cell dissemination into the peritoneal cavity through the fallopian tubes. Seven included studies reported PPC. Six of these studies (15–17, 19, 21, 22) suggested that no significant difference was found between patients who did or did not undergo hysteroscopy based on PPC in early-stage endometrial cancer, but the low risk of disease (such as G1/G2 and Type I), low infusion pressure of hysteroscopy, and small numbers of patients might have influenced the results (15, 16). In contrast, one of these seven studies (18) reported that there was a significant difference between groups and the authors hypothesized that the aggressive nature of type II endometrial cancer might contribute to this (p=0.002). Some studies (8, 24, 25) also demonstrated that hysteroscopy might increase the risk of PPC in early-stage endometrial cancer. However, a meta-analysis by Chang et al. (26) proved that diagnostic hysteroscopy might increase the risk of PPC, but not when the tumor was in an early stage (I or II).

Although the relationship between PPC and hysteroscopy is controversial, the real concern about early-stage endometrial cancer is whether PPC caused by hysteroscopy would result in worse survival. Some studies (23, 27) suggested that PPC was not an independent prognostic factor for early-stage endometrial cancer and was not related to a worse prognosis, while other studies (28–30) demonstrated that PPC was independently associated with decreased DFS, DDS, and OS in early-stage endometrial cancer. A meta-analysis by Lee et al. (31) suggested that PPC might be a potential prognostic factor for early-stage endometrial cancer because it was associated with other prognostic factors, such as histology type, myometrial invasion, and surgical stage.

Gurkan et al. (9) assessed the dissemination of malignant cells caused by hysteroscopy in an *in vitro* model and suggested that these cells could be functionally viable and adhere to a matrix. However, Neis et al. (32) detected a single tumor-cell complex in a part of the fallopian tube in only one patient among 118 patients. It is possible that the receipt of adjuvant therapy, the low concentration of cancer cells in the peritoneal cavity, and the unsuitable tumor microenvironment *in vivo* limited the malignant behavior and progression of malignant cells. Therefore, the cells eventually die. Further studies are needed to confirm this hypothesis.

There are some limitations to this study. First, the number of patients included in the meta-analysis depending on 5-year DFS may not be sufficient to draw definitive conclusions (600 patients in the hysteroscopy group and 1225 patients in the non-hysteroscopy group). Second, six of eight studies included in this study were retrospective studies. Inherent biases in observational studies, such as selection bias, could not be avoided. Third, the number of studies included in the meta-analysis based on 5-year OS and DSS was small, leading to significant heterogeneity. Fourth, the possible heterogeneity of different adjuvant treatment between the included studies is still a limitation. Lastly, the follow-up time ranging from 1.13-152 months might not be long enough to observe the number of deaths. Thus, more prospective RCTs with enough observation time are needed.

## Conclusion

This is the first systematic review and meta-analysis to evaluate the safety of hysteroscopy in early-stage endometrial cancer. The results indicate that hysteroscopy is not associated with worse prognosis in patients with early-stage endometrial cancer. This finding supports previous conclusions that hysteroscopy is a safe diagnostic method. Nonetheless, more RCTs are needed to confirm this report.

## Data Availability Statement

The original contributions presented in the study are included in the article/**Supplementary Material**. Further inquiries can be directed to the corresponding authors.

## Author Contributions

YD, conceptualization, investigation, writing-original draft, writing - review and editing. YX, conceptualization, investigation, writing-review and editing. ZQ, conceptualization, investigation, writing- review and editing. LS conceptualization, investigation, writing-review and editing. YC, LH, and AZ, final approval of the version, writing- review and editing. All authors contributed to the article and approved the submitted version.

## Funding

This work was supported by the project of Scientific and Technological Department of Sichuan Province (Project No: 2019YFS0417).

## Conflict of Interest

The authors declare that the research was conducted in the absence of any commercial or financial relationships that could be construed as a potential conflict of interest.

## Publisher’s Note

All claims expressed in this article are solely those of the authors and do not necessarily represent those of their affiliated organizations, or those of the publisher, the editors and the reviewers. Any product that may be evaluated in this article, or claim that may be made by its manufacturer, is not guaranteed or endorsed by the publisher.
