# Supplementary material for: The Oncology Safety of Diagnostic Hysteroscopy in Early-Stage Endometrial Cancer: A Systematic Review and Meta-Analysis
Source: Front Oncol. 2021 Oct 21;11:742761. doi: 10.3389/fonc.2021.742761 (PMC8566916; doi:10.3389/fonc.2021.742761)
Supplement: Supplementary file 1 [file Image_1.pdf]

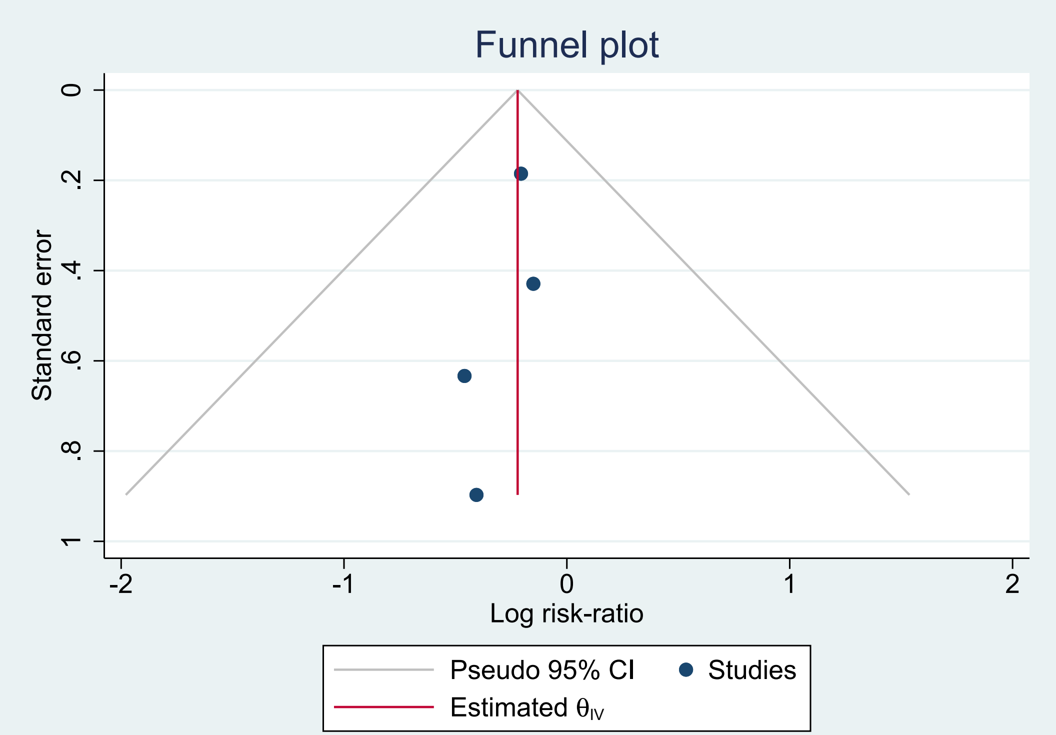

Supplementary 2 Figure1.Funnel plot of trials on oncology effect of hysteroscopy in early-stage endometrial cancer.
