# Supplementary material for: The Oncology Safety of Diagnostic Hysteroscopy in Early-Stage Endometrial Cancer: A Systematic Review and Meta-Analysis
Source: Front Oncol. 2021 Oct 21;11:742761. doi: 10.3389/fonc.2021.742761 (PMC8566916; doi:10.3389/fonc.2021.742761)
Supplement: Supplementary file 2 [file Table_1.docx]

S1. The details of search strategies.

| Database | Search step | Search strategy | Search result |
| --- | --- | --- | --- |
| PubMed | #1 | Endometrial Neoplasms[Mesh] | 21,827 |
|  | #2 | ("endometrial"[Title/Abstract] OR "endometrium"[Title/Abstract]) AND ("cancer*"[Title/Abstract] OR "carcinoma*"[Title/Abstract]) | 36,970 |
|  | #3 | (#1 OR #2) | 38,161 |
|  | #4 | Hysteroscopy"[Mesh] | 4,824 |
|  | #5 | Hysteroscop* | 7,025 |
|  | #6 | #4 OR #5 | 8,001 |
|  | #7 | #3 AND #6 | 1,283 |
|  | #8 | ("randomized controlled trial[publication type]" OR "controlled clinical trial"[publication type] OR "clinical trials as topic" [Mesh:NoExp] OR randomized[Title/Abstract] OR placebo[Title/Abstract] OR randomly[Title/Abstract] OR trial[Title/Abstract])NOT("Animals"[Mesh] NOT "Humans"[Mesh]) | 2,689,922 |
|  | #9 | cohort studies[Mesh:NoExp] OR longitudinal studies[Mesh:NoExp] OR follow-up studies[Mesh:NoExp] OR prospective studies[Mesh:NoExp] OR retrospective studies[Mesh:NoExp] OR cohort[Title/Abstract] OR longitudinal[Title/Abstract] OR prospective[Title/Abstract] OR retrospective[Title/Abstract] | 1,391,653 |
|  | #10 | #8 OR #9 | 3,747,494 |
|  | #11 | #7 AND #11 | 548 |
| Embase | #1 | 'endometrium cancer'/exp | 49908 |
|  | #2 | (endometrial:ab,ti OR endometium:ab,ti) AND (cancer* OR carcinoma* ) | 50,791 |
|  | #3 | (#1 OR #2) | 12,114 |
|  | #4 | 'hysteroscopy'/exp | 397,806 |
|  | #5 | hysteroscop*:ab,ti | 11,916 |
|  | #6 | #4 OR #5 | 147,621 |
|  | #7 | #6 AND #3 | 2612 |
|  | #8 | 'crossover procedure':de OR 'double-blind procedure':de OR 'randomized controlled trial':de OR 'single-blind procedure':de OR random*:de,ab,ti OR factorial*:de,ab,ti OR crossover*:de,ab,ti OR ((cross NEXT/1 over*):de,ab,ti) OR placebo*:de,ab,ti OR ((doubl* NEAR/1 blind*):de,ab,ti) OR ((singl* NEAR/1 blind*):de,ab,ti) OR assign*:de,ab,ti OR allocat*:de,ab,ti OR volunteer*:de,ab,ti | 2615,035 |
|  | #9 | 'cohort analysis'/exp OR 'longitudinal study'/exp OR 'prospective study'/exp OR 'follow up'/exp OR 'cohort$':ab,ti | 2,926,690 |
|  | #10 | #8 OR #9 | 5,113,747 |
|  | #11 | #7 AND #11 | 738 |
| Cochrane | #1 | MeSH descriptor: [Endometrial Neoplasms] explode all trees        1921 | 170,912 |
|  | #2 | (endometrial:ti,ab,kw or endometrium:ti,ab,kw) AND (cancer*:ti,ab,kw OR carcinoma*:ti,ab,kw | 2,452 |
|  | #3 | #1 OR #2 | 1,082,632 |
|  | #4 | MeSH descriptor: [hysteroscopy] explode all trees | 399 |
|  | #5 | (Hysteroscop*):ti,ab,kw | 1,921 |
|  | #6 | #4 OR #5 | 1,521 |
|  | #7 | #3 AND #6 | 399 |
